# Supplementary material for: Evaluation of 6-OxP-CD, an Oxime-based cyclodextrin as a viable medical countermeasure against nerve agent poisoning: Experimental and molecular dynamic simulation studies on its inclusion complexes with cyclosarin, soman and VX
Source: PLoS One. 2023 Mar 30;18(3):e0283181. doi: 10.1371/journal.pone.0283181 (PMC10062596; doi:10.1371/journal.pone.0283181)
Supplement: S6 File — (DOCX) [file pone.0283181.s006.docx]

**-Supporting Information-**

**Evaluation of 6-OxP-CD, an Oxime-based Cyclodextrin as a Viable Medical Countermeasure Against Nerve Agent Poisoning: Experimental and Molecular Dynamic Simulation Studies on Its Inclusion Complexes with Cyclosarin, Soman and VX**

Edmond Y. Lau^1,^*, Heather A. Enright^1^, Victoria Lao^1^, Michael A. Malfatti^1^, Brian P. Mayer^2-4^, Audrey M. Williams^2-4^, Carlos A. Valdez^2-4,^*

*^1^Biosciences and Biotechnology Division,^2^Global Security Directorate, ^3^Nuclear and Chemical Sciences Division ^4^Forensic Science Center, Lawrence Livermore National Laboratory, Livermore, CA, 94550, United States.*

**Table of MM/GBSA energies for Cyclosarin and Soman with 6-OxP-CD**

MM/GBSA energies Cyclosarin-6-OxP-CD, values are in kcal/mole and standard deviations are in parenthesis


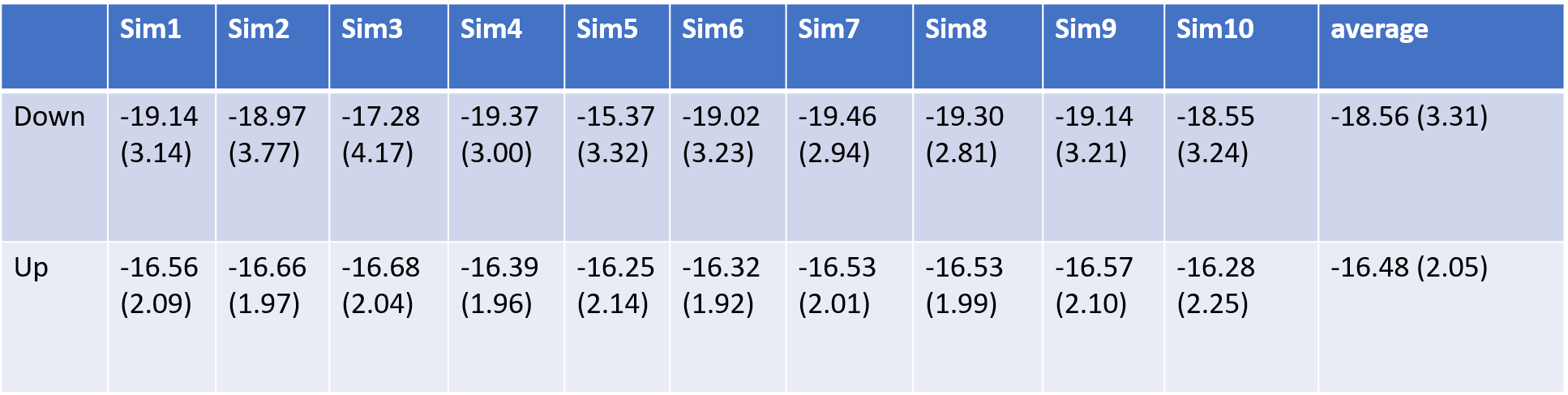


MM/GBSA energies Soman-6-OxP-CD, values in kcal/mole and standard deviations are in parenthesis


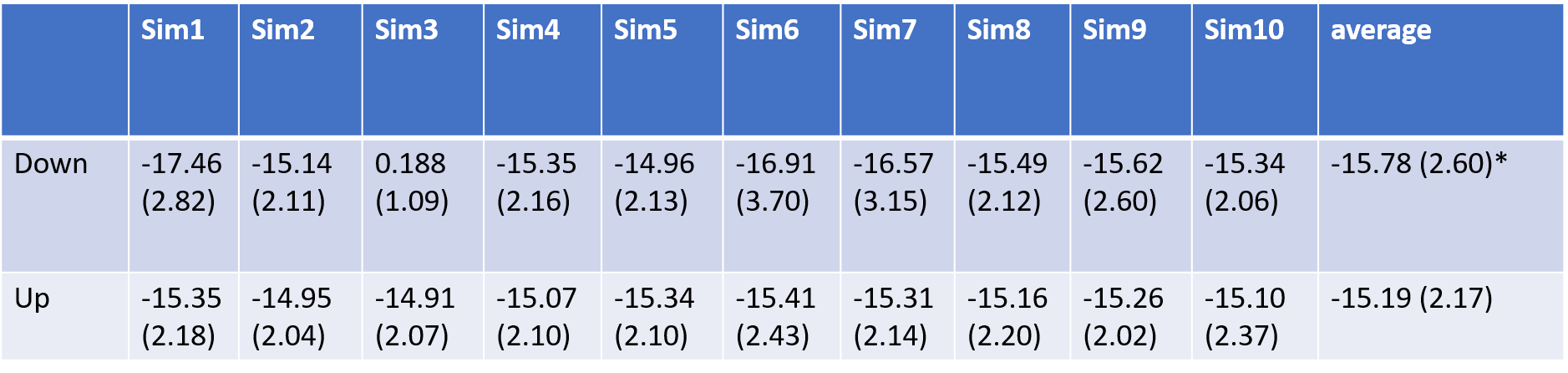


*Average calculated without Sim3 (soman diffused out of the cyclodextrin).

**Topology files for molecules employed in calculations**

**Oxime**

@<TRIPOS>MOLECULE

OXN

14 14 1 0 0

SMALL

bcc

@<TRIPOS>ATOM

1 C 0.0490 0.0000 0.0320 ca 1 OXN -0.172300

2 N -0.0010 0.0020 1.3720 nb 1 OXN 0.111900

3 C1 1.1210 0.0020 2.1490 ca 1 OXN -0.238300

4 C2 2.3640 0.0000 1.5160 ca 1 OXN -0.092000

5 C3 2.4550 -0.0030 0.1350 ca 1 OXN -0.157100

6 C4 1.2810 -0.0030 -0.6820 ca 1 OXN 0.013100

7 C5 1.3060 -0.0060 -2.0920 c2 1 OXN 0.072800

8 N1 0.1360 -0.0060 -2.7660 n2 1 OXN -0.115700

9 O 0.2040 -0.0080 -4.0250 o 1 OXN -0.283000

10 H 0.9760 0.0040 3.2210 h4 1 OXN 0.168000

11 H1 3.2590 0.0000 2.1290 ha 1 OXN 0.151300

12 H2 3.4290 -0.0050 -0.3450 ha 1 OXN 0.153300

13 H3 -0.8900 0.0000 -0.5070 h4 1 OXN 0.189000

14 H4 2.2500 -0.0080 -2.6420 h4 1 OXN 0.002800

@<TRIPOS>BOND

1 1 2 ar

2 1 6 ar

3 1 13 1

4 2 3 ar

5 3 4 ar

6 3 10 1

7 4 5 ar

8 4 11 1

9 5 6 ar

10 5 12 1

11 6 7 1

12 7 8 2

13 7 14 1

14 8 9 1

@<TRIPOS>SUBSTRUCTURE

1 OXN 1 TEMP 0 **** **** 0 ROOT

**Cyclosarin**

@<TRIPOS>MOLECULE

CSA

25 25 1 0 0

SMALL

bcc

@<TRIPOS>ATOM

1 C 1.1606 7.4073 0.9948 c3 1 CSA 0.1831

2 C1 2.6603 7.2634 1.2379 c3 1 CSA -0.0894

3 O 0.9794 7.6530 -0.4367 os 1 CSA -0.6142

4 C2 2.9422 6.9184 2.7104 c3 1 CSA -0.0764

5 H 3.0438 6.4667 0.5863 hc 1 CSA 0.0500

6 H1 3.1699 8.1902 0.9480 hc 1 CSA 0.0500

7 C3 2.1751 5.6629 3.1518 c3 1 CSA -0.0794

8 H2 4.0207 6.7812 2.8562 hc 1 CSA 0.0442

9 H3 2.6478 7.7671 3.3452 hc 1 CSA 0.0442

10 C4 0.6688 5.8081 2.8841 c3 1 CSA -0.0764

11 H4 2.5587 4.7918 2.6019 hc 1 CSA 0.0422

12 H5 2.3530 5.4658 4.2170 hc 1 CSA 0.0422

13 C5 0.3883 6.1551 1.4115 c3 1 CSA -0.0894

14 H6 0.1395 4.8852 3.1522 hc 1 CSA 0.0442

15 H7 0.2592 6.5992 3.5290 hc 1 CSA 0.0442

16 H8 0.7015 5.3251 0.7634 hc 1 CSA 0.0500

17 H9 -0.6848 6.3045 1.2453 hc 1 CSA 0.0500

18 H10 0.7769 8.2818 1.5325 h1 1 CSA 0.0757

19 P -0.2208 8.5766 -0.9672 p5 1 CSA 1.5993

20 C6 0.3150 8.9855 -2.6410 c3 1 CSA -0.4593

21 O1 -0.6609 9.6789 -0.0851 o 1 CSA -0.8423

22 F -1.3862 7.5174 -1.2484 f 1 CSA -0.3414

23 H11 -0.5073 9.4681 -3.1741 hc 1 CSA 0.1167

24 H12 1.1598 9.6777 -2.5882 hc 1 CSA 0.1167

25 H13 0.6187 8.0831 -3.1781 hc 1 CSA 0.1167

@<TRIPOS>BOND

1 1 2 1

2 1 3 1

3 1 13 1

4 1 18 1

5 2 4 1

6 2 5 1

7 2 6 1

8 3 19 1

9 4 7 1

10 4 8 1

11 4 9 1

12 7 10 1

13 7 11 1

14 7 12 1

15 10 13 1

16 10 14 1

17 10 15 1

18 13 16 1

19 13 17 1

20 19 20 1

21 19 21 2

22 19 22 1

23 20 23 1

24 20 24 1

25 20 25 1

@<TRIPOS>SUBSTRUCTURE

1 CSA 1 TEMP 0 **** **** 0 ROOT

**Soman**

@<TRIPOS>MOLECULE

SOM

27 26 1 0 0

SMALL

bcc

@<TRIPOS>ATOM

1 C 2.1417 6.2369 1.9801 c3 1 SOM -0.0580

2 C1 1.9450 5.4683 0.6585 c3 1 SOM -0.0891

3 C2 3.6399 6.2480 2.3405 c3 1 SOM -0.0891

4 C3 1.3435 5.5201 3.0889 c3 1 SOM -0.0891

5 C4 1.5904 7.6787 1.8130 c3 1 SOM 0.2131

6 H 3.8225 6.7074 3.3175 hc 1 SOM 0.0394

7 H1 4.0223 5.2220 2.3841 hc 1 SOM 0.0394

8 H2 4.2210 6.7965 1.5923 hc 1 SOM 0.0394

9 H3 1.5168 5.9622 4.0749 hc 1 SOM 0.0394

10 H4 0.2642 5.5442 2.8905 hc 1 SOM 0.0394

11 H5 1.6451 4.4684 3.1433 hc 1 SOM 0.0394

12 H6 0.8854 5.4350 0.3696 hc 1 SOM 0.0394

13 H7 2.5095 5.9266 -0.1577 hc 1 SOM 0.0394

14 H8 2.2888 4.4333 0.7650 hc 1 SOM 0.0394

15 H9 0.5390 7.6009 1.5057 h1 1 SOM 0.0087

16 C5 1.6870 8.5886 3.0351 c3 1 SOM -0.1221

17 O 2.3422 8.3100 0.7258 os 1 SOM -0.5962

18 H10 1.2615 9.5669 2.7999 hc 1 SOM 0.0530

19 H11 1.1291 8.1703 3.8783 hc 1 SOM 0.0530

20 H12 2.7278 8.7309 3.3372 hc 1 SOM 0.0530

21 P 1.7295 9.3012 -0.3743 p5 1 SOM 1.6053

22 O1 2.7319 10.1682 -1.0164 o 1 SOM -0.8113

23 C6 0.6899 8.3202 -1.4854 c3 1 SOM -0.4803

24 F 0.6117 10.0934 0.4573 f 1 SOM -0.3504

25 H13 0.1488 8.9946 -2.1548 hc 1 SOM 0.1154

26 H14 1.3279 7.6660 -2.0858 hc 1 SOM 0.1154

27 H15 -0.0296 7.7117 -0.9316 hc 1 SOM 0.1154

@<TRIPOS>BOND

1 1 2 1

2 1 3 1

3 1 4 1

4 1 5 1

5 2 12 1

6 2 13 1

7 2 14 1

8 3 6 1

9 3 7 1

10 3 8 1

11 4 9 1

12 4 10 1

13 4 11 1

14 5 15 1

15 5 16 1

16 5 17 1

17 16 18 1

18 16 19 1

19 16 20 1

20 17 21 1

21 21 22 2

22 21 23 1

23 21 24 1

24 23 25 1

25 23 26 1

26 23 27 1

@<TRIPOS>SUBSTRUCTURE

1 SOM 1 TEMP 0 **** **** 0 ROOT

**VX**

@<TRIPOS>MOLECULE

VXC

43 42 1 0 0

SMALL

bcc

@<TRIPOS>ATOM

1 C -2.0067 8.7413 -1.3186 c3 1 VXC -0.4693

2 P -0.2835 8.1822 -1.1693 p5 1 VXC 1.4253

3 O 0.7385 9.2668 -1.0734 o 1 VXC -0.7673

4 O1 -0.0764 7.0657 -2.3158 os 1 VXC -0.5872

5 C1 0.0829 7.4591 -3.7189 c3 1 VXC 0.1604

6 C2 1.5515 7.5494 -4.0879 c3 1 VXC -0.1191

7 S -0.2501 6.9181 0.5718 ss 1 VXC -0.6455

8 C3 1.1720 7.6884 1.4541 c3 1 VXC 0.1447

9 C4 2.4501 6.8492 1.4837 c3 1 VXC 0.1148

10 H -0.4334 6.6794 -4.2827 h1 1 VXC 0.0752

11 H1 -0.4323 8.4091 -3.9009 h1 1 VXC 0.0752

12 H2 2.0568 6.5987 -3.8897 hc 1 VXC 0.0514

13 H3 1.6481 7.7732 -5.1568 hc 1 VXC 0.0514

14 H4 2.0465 8.3430 -3.5207 hc 1 VXC 0.0514

15 H5 1.4004 8.6072 0.9007 h1 1 VXC 0.0842

16 H6 0.8366 7.9789 2.4520 h1 1 VXC 0.0842

17 H7 2.5203 6.2160 0.6011 hx 1 VXC 0.1132

18 H8 3.3199 7.5078 1.4778 hx 1 VXC 0.1132

19 H9 -2.1389 9.2473 -2.2809 hc 1 VXC 0.1500

20 H10 -2.2260 9.4506 -0.5164 hc 1 VXC 0.1500

21 H11 -2.6948 7.8933 -1.2620 hc 1 VXC 0.1500

22 N 2.6692 5.9341 2.6946 n4 1 VXC -0.6834

23 H12 3.6297 5.6017 2.5544 hn 1 VXC 0.4338

24 C5 2.7216 6.6862 4.0462 c3 1 VXC 0.1380

25 C6 1.8480 4.6062 2.7396 c3 1 VXC 0.1380

26 C7 3.2170 5.7486 5.1529 c3 1 VXC -0.1333

27 C8 3.6127 7.9278 3.9469 c3 1 VXC -0.1333

28 H13 1.6915 6.9868 4.2457 hx 1 VXC 0.0952

29 H14 3.6934 8.3681 4.9444 hc 1 VXC 0.0758

30 H15 4.6298 7.6757 3.6224 hc 1 VXC 0.0758

31 H16 3.2109 8.7006 3.2877 hc 1 VXC 0.0758

32 H17 2.4262 4.0029 3.4434 hx 1 VXC 0.0952

33 C9 0.4325 4.7739 3.2925 c3 1 VXC -0.1333

34 C10 1.8885 3.9035 1.3821 c3 1 VXC -0.1333

35 H18 1.5319 2.8785 1.5264 hc 1 VXC 0.0758

36 H19 1.2378 4.3798 0.6438 hc 1 VXC 0.0758

37 H20 2.9061 3.8341 0.9802 hc 1 VXC 0.0758

38 H21 3.3096 6.3267 6.0764 hc 1 VXC 0.0758

39 H22 2.5373 4.9200 5.3605 hc 1 VXC 0.0758

40 H23 4.2106 5.3440 4.9227 hc 1 VXC 0.0758

41 H24 -0.0256 3.7807 3.3292 hc 1 VXC 0.0758

42 H25 0.4246 5.1664 4.3122 hc 1 VXC 0.0758

43 H26 -0.2112 5.3977 2.6642 hc 1 VXC 0.0758

@<TRIPOS>BOND

1 1 2 1

2 1 19 1

3 1 20 1

4 1 21 1

5 2 3 2

6 2 4 1

7 2 7 1

8 4 5 1

9 5 6 1

10 5 10 1

11 5 11 1

12 6 12 1

13 6 13 1

14 6 14 1

15 7 8 1

16 8 9 1

17 8 15 1

18 8 16 1

19 9 17 1

20 9 18 1

21 9 22 1

22 22 23 1

23 22 24 1

24 22 25 1

25 24 26 1

26 24 27 1

27 24 28 1

28 25 32 1

29 25 33 1

30 25 34 1

31 26 38 1

32 26 39 1

33 26 40 1

34 27 29 1

35 27 30 1

36 27 31 1

37 33 41 1

38 33 42 1

39 33 43 1

40 34 35 1

41 34 36 1

42 34 37 1

@<TRIPOS>SUBSTRUCTURE

1 VXC 1 TEMP 0 **** **** 0 ROOT
