# Supplementary material for: Evaluation of 6-OxP-CD, an Oxime-based cyclodextrin as a viable medical countermeasure against nerve agent poisoning: Experimental and molecular dynamic simulation studies on its inclusion complexes with cyclosarin, soman and VX
Source: PLoS One. 2023 Mar 30;18(3):e0283181. doi: 10.1371/journal.pone.0283181 (PMC10062596; doi:10.1371/journal.pone.0283181)
Supplement: Attachment — Submitted filename: Point_by_point_responses_requested_02022023.docx [file pone.0283181.s007.docx]

**Point by Point Responses to Reviewers**

Dear Reviewers,

We would like to thank you for the careful review of our manuscript. We have made many changes to the original version. Aside from addressing your questions, we have added all our experimental work with the 6-OxP-CD system. This work includes all the NMR data collected for the CD against the three nerve agents discussed in the original version of the manuscript. These additions are highlighted in the new version of the manuscript, and we have done our best effort to add the experimental work to support all the computational work explained in the original version.

We hope our responses are clear and if there is anything not clear, do not hesitate to let us know.

Best,

The authors

**Review Comments to the Author**

**Reviewer #1:** This is an interesting paper that uses molecular dynamics to predict the ability of 6-OxP-CD to bind and react with 3 organophosphorus (OP) nerve agents (NAs): GF, GD, and VX. This paper appears to have been stimulated by the observation (ref. 32) that 6-OxP-CD efficiently degraded GF. The computational analysis here rationalized this point, showing close proximity of GF to one orientation (the “down” position) of 6-OxP-CD. The authors then undertook computational analysis of two other OPs, GD and VX. GD appeared to bind 6-OxP-CD in both the “down” and “up” positions. The simulated binding time was quite long in the “up” position where the P atom and the oxime were very far apart. This orientation was proposed to compete successfully with the complex in the “down” position, effectively rationalizing the much slower degradation rate for GD. Additional simulations of VX and 6-OxP-CD failed to show significant binding or reactivity.

The reviewer is not an expert in molecular dynamics simulations, but they seem sound. One comment should be considered for inclusion in the “Conclusions” section:
While not expected in this manuscript, can molecular dynamics be used to make predictions about alternatives to the 6-OxP-CD structure evaluated here? In particular, are isomers available with the 6-Ox moiety or is the CD structure (and its available -OH groups) completely symmetrical? If there is complete symmetry, could one consider a second substituent that would make isomers worth consideration?

**Response:** Yes, molecular dynamics can certainly be used to evaluate alternative isomers for 6-OxP-CD scaffold. In fact, the original work (Zeggerle *et al.*) experimentally evaluated the other two isomers of 6-OxP-CD, that is the *ortho*- and the *para*-oxime isomers. These two other isomers were found to be ineffective in degrading Cyclosarin (GF). At this point we do not know if these other two isomers would be effective against Soman or VX as we did not synthesize these and thus, their syntheses laid outside the scope of this specific paper. Current work is focusing in on the analysis of such isomers against these agents, we’re hoping to find some potentially effective candidates for this application.

**Reviewer #2:** This manuscript reports the structural analysis of Molecular Dynamics simulations performed for complexes of an oxime-based cyclodextrin (6-OxP-CD) with three nerve agents: cyclosarin, soman and VX. Although the topic has attracted much interest in the literature, gaining insight into the binding and degradation mechanism by derivatized cyclodextrins deserves interest. However, this study seems preliminary and publication at this stage is premature for several reasons.

1) The authors state that they have performed MM-GBSA calculations to estimate the binding free energy. But the results of these computations are not included in the manuscript.

***Response****:* We have significantly modified the manuscript by adding our experimental work to it.

While MM-GBSA calculations could be a valuable ingredient to complement this study, the lack of results (and a detailed discussion about the reliability of MM_GBSA binding affinities) raises serious doubts about some conclusion stated at the end of the manuscript. For instance,
i) regarding the complex with cyclosarin, it is stated that the 'up binding mode', which is not suitable for degradation, is 'energetically favored' relative to the 'down binding mode'. If the difference in binding affinity is substantial, cyclosarin could bind the cyclodextrin, but would never be degraded. How can this be reconciled with the experimental observation that cyclosarin is degraded instantaneously?

***Response***: We apologize for the misprint. It should be stated that the up binding mode is higher in energy than the down binding mode although the with standard deviations the values overlap. The text has been updated to reflect this correction.

MM/GBSA energies Cyclosarin-6-OxP, values are in kcal/mole and standard deviations are in parenthesis.

ii) regarding the complex with soman, the text states that the 'up binding mode' forms 'a stable enough complex to compete with the 'down-GD' conformation.' Again, without quantitative data, this statement is vague and has little usefulness for the discussion.

***Response***: For soman, the MM/GBSA energies for the up binding mode is virtually identical to the down binding mode (without the contribution from Sim3 (down)). The manuscript has been updated with these results. Both tables will be added to the supplementary materials.

MM/GBSA energies Soman-6-OxP, values in kcal/mole and standard deviations are in parenthesis

*Average calculated without Sim3 (soman diffused out of the cyclodextrin).

2) Several key points of the manuscript are directly related to experimental results obtained by the authors that are not included in the study: reference 34 (unpublished results), which is quoted in pages 6 and 8, the half-life times reported in Conclusions (page 16), or the NMR data collected for assays with VX that support the lack of binding (reported as data not shown in page 15). This is a serious weakness, because the experimental results are fundamental to fully understand the impact of present simulations. In my view, the impact of this study would be much higher if both experimental and computational studies are presented together in a single manuscript.

***Response****:* We have significantly modified the manuscript by adding our experimental work to it.

3) A number of technical details are confusing. For example,
i) while the text states that simulations were performed for a total of 10 or 30 ns depending on the nerve agent (page 7), plots of distances correspond to simulations of 50 ns.

***Response:*** Some of the simulations were extended out to 50 ns to investigate the conformations taken between the agent and the 6-OxP. We’ve added a sentence to indicate in the text.

ii) similarly, it seems that replicate simulations were performed for MM-GBSA simulations in order to obtain averaged estimates (page 7), but this is not described subsequently in the manuscript.

***Response:*** We have included the Tables in the supplementary materials for the MM/GBSA energies.

iii) more importantly, models were derived for the oxime in ionized and unionized forms (page 9), but results are reported only for the former species. Is the ionized form the most populated in aqueous solution? If not, can the proton be released during the chemical attack to the phosphorus atom? This should be discussed in the text due to the mechanistic implications. Furthermore, the final force field parameters should be provided as Supporting Information.

***Response:*** Although it has not been conclusively determined if the catalytic form of the oxime is ionized or unionized, experimentally there has been compelling support for the ionized form of the oxime (oximate). We have also included the force field parameters in the supplementary materials.

*Detoxification of tabun at physiological pH mediated by substituted β-cyclodextrin and glucose derivatives containing oxime groups*

Florian Brandhuber, Michael Zengerle, Luzian Porwol, Oliver Tenberken, Horst Thiermann, Franz Worek, Stefan Kubik, Georg Reiter

Toxicology. 2012 Dec 16;302(2-3):163-71. doi: 10.1016/j.tox.2012.08.013. Epub 2012 Sep 11.

*Highly efficient cyclosarin degradation mediated by a β-cyclodextrin derivative containing an oxime-derived substituent*

Michael Zengerle, Florian Brandhuber, Christian Schneider, Franz Worek, Georg Reiter, Stefan Kubik

Beilstein J Org Chem. 2011;7:1543-54. doi: 10.3762/bjoc.7.182. Epub 2011 Nov 22.

*Amino acid residues controlling reactivation of organophosphonyl conjugates of acetylcholinesterase by mono- and bisquaternary oximes*

Y Ashani, Z Radić, I Tsigelny, D C Vellom, N A Pickering, D M Quinn, B P Doctor, P Taylor

J Biol Chem. 1995 Mar 17;270(11):6370-80. doi: 10.1074/jbc.270.11.6370.
